# Widespread emergence of *Staphylococcus aureus* with variant FarR regulators and enhanced resistance to antimicrobial fatty acids within clonal complex CC5, CC8, and CC97 strains from human and bovine hosts

**DOI:** 10.1128/spectrum.02278-25

**Published:** 2025-10-31

**Authors:** Camryn M. Bonn-Dunbar, Idowu B. Olawoye, Akanksha Thukral, Jennifer L. Guthrie, Martin J. McGavin

**Affiliations:** 1 Department of Microbiology and Immunology, University of Western Ontario 6221, London, Ontario, Canada; 2 Schulich School of Medicine and Dentistry, University of Western Ontario 6221, London, Ontario, Canada; University of Manitoba, Winnipeg, Canada

**Keywords:** *Staphylococcus aureus*, MRSA, antimicrobial fatty acid, efflux pumps, phylogenetic analysis

## Abstract

The FarER efflux pump and regulator confer inducible resistance of *Staphylococcus aureus* to linoleic acid, an unsaturated antimicrobial fatty acid that comprises a chemical component of innate immunity at sites of colonization and infection. We previously discovered *farR* through *in vitro* selection for increased resistance to linoleic acid, leading to recovery of FarR^H121Y^ that promoted enhanced expression of *farE*. Here, we report on the widespread emergence of increased resistance to linoleic acid across the phylogenetic spectrum of *S. aureus* through mechanisms that are both dependent and independent of *farER*. In this latter group, sequence type ST228 healthcare-associated methicillin-resistant *S. aureus* has FarR^A14P^ that is defective in DNA binding and cannot induce *farE* expression, and yet these strains exhibit enhanced resistance to linoleic acid. In the former group, we note the polyphyletic emergence of FarR^G166D^ in ST5 community-acquired MRSA (CA-MRSA), adding to previously noted variants FarR^C116Y^ and FarR^E160G^ in ST5 MRSA. We also observe FarR^H121Y^ and FarR^G166D^ in ST97 methicillin-susceptible *S. aureus* from bovine hosts, together with polyphyletic emergence of FarR^C116Y^. Last, we note the emergence of FarR^H121Y^ in the USA300 lineage of ST8 CA-MRSA from the UK, in association with an SCC*mec* VI element that encodes resistance to fusidic acid, a topical antimicrobial used to treat superficial skin infections. Our data support the widespread emergence and dissemination of *S. aureus* strains from both human and bovine hosts, with enhanced resistance to unsaturated free fatty acids that comprise an important chemical component of innate immunity.

**IMPORTANCE:**

*Staphylococcus aureus* is a priority pathogen for the World Health Organization due to its association with human morbidity, mortality, and acquisition of multiple drug resistance. In addition to acquiring specific antimicrobial resistance genes, it is also widely documented that *S. aureus* can evolve through accumulation of genetic polymorphisms that confer reduced susceptibility to some antibiotics such as daptomycin and vancomycin. In this study, we report on the widespread emergence of *S. aureus* strains with sufficiency for increased resistance to antimicrobial fatty acids through polymorphisms in the regulator of an efflux pump. This is an indication that, in addition to evolution in response to antibiotics that are used to treat infections, *S. aureus* also exhibits the capacity to evolve in response to antimicrobial unsaturated fatty acids that are important chemical components of innate immunity. These findings underscore the exceptional capacity of *S. aureus* to evolve in response to antimicrobial threats.

## INTRODUCTION

*Staphylococcus aureus* has emerged as and remained a leading cause of skin and soft-tissue infections (SSTIs) (1, 2), accumulating severe financial and healthcare burdens (3). Further implications include concurrent SSTI as a potential indicator of subsequent bloodstream infections (BSI) (4), where BSI typically cause significant mortality (5, 6). The majority of *S. aureus* infections in North America are attributed to a community-acquired methicillin-resistant *S. aureus* (CA-MRSA) clone designated as USA300, which is multilocus sequence type ST8 (4, 7, 8). CA-MRSA infections are often acquired from the colonizing strain (8), emphasizing the role of colonization in predicting subsequent infection and morbidity. On average, *S. aureus* asymptomatically colonizes 30% of healthy adults in the anterior nares (9, 10). One host-derived mechanism to promote antibacterial activity and combat *S. aureus* colonization is the production of long-chain antimicrobial fatty acids at sites of colonization in the anterior nares and on skin (11, 12). Using a healthcare-associated MRSA (HA-MRSA) strain, MRSA252, to assess global changes in gene expression in response to linoleic acid, several genes that were upregulated and spanning a range of metabolic, regulatory, efflux, and cell envelope-related functions were found to contribute to resistance, alluding to a multifactorial process (13). One of these genes, SAR2632, defined as an MmpL family efflux pump, was also identified in our own work through *in vitro* selection for increased resistance of *S. aureus* USA300 to linoleic acid, leading to our description of the Resistance-Nodulation-Division (RND) superfamily efflux pump FarE and divergently transcribed TetR family regulator (TFR) FarR, which confer resistance to unsaturated free fatty acids (uFFA) (13).

In gram-negative bacteria, the RND superfamily of efflux pumps is a critical mechanism of resistance to a broad range of antimicrobial agents, including antibiotics (14), dyes (15), heavy metals (16), and lipids such as bile salts (17) or fatty acids (18). Although RND pumps may be regulated by a variety of global and local regulators (19, 20), the most common is the TFR, which typically represses a cognate divergently transcribed RND efflux pump (21). Consequently, mutations that inactivate expression or disrupt DNA binding of TFRs typically promote enhanced expression of the co-associated RND efflux pump, concomitant with enhanced resistance (22, 23). However, the role of TFRs in modulating RND pumps and their *in vivo* impact on resistance phenotypes is less well established in gram-positive microbes. Indeed, although the *farR-farE* regulator and efflux pump in *S. aureus* maintain the divergent transcription arrangement that is common in gram-negative bacteria, transposon-mediated insertional inactivation of *farR* led to loss of inducible expression of *farE* (13), deviating from the canonical paradigm of TFR repressors, such that emergence of enhanced resistance should not occur through inactivating mutations in FarR.

Although our previous work implicated FarR as being essential for expression of FarE, we nevertheless discovered FarR and FarE through *in vitro* selection for increased resistance to linoleic acid in the USA300 ST8 CA-MRSA, from which we identified a FarR^H121Y^ variant that promoted increased expression of FarE in a strain that we designated FAR7 (13). We subsequently observed multiple emergences of FarR variants in the USA100 lineage of ST5 HA-MRSA, including FarR^E93EE^, FarR^C116Y^, FarR^E160G^, FarR^P165L^, and FarR^G166D^, which collectively accounted for 5.7% of the total *S. aureus* ST5 genomes that have been sequenced. Of these, FarR^C116Y^ and Far^E160G^ were sufficient to promote increased resistance to linoleic acid. Evidence of positive selection was supported through the polyphyletic emergence of FarR^C116Y^ in ST5 HA-MRSA from Canada and the United States, and there were also separate emergences of FarR^G166D^ in Europe and the United States, although we did not assess strains with this latter variant (24). Although FarR^H121Y^, which we discovered through *in vitro* selection, was not evident in ST5 HA-MRSA, we noted its appearance among ST97 *S. aureus*, together with FarR^C116Y^ and FarR^G166D^. ST97 *S. aureus* are known to cause infection in human hosts as well as being a major cause of mastitis in dairy cattle (25–28). Moreover, as more genome sequences have become available, we have now noted the emergence of USA300 CA-MRSA strains with FarR^H121Y^ in the UK.

We now report new findings on the emergence, phylogenetics, and resistance phenotype of *S. aureus* strains with variant FarR regulators that were not evident in our initial focus on the USA100 lineage of ST5 HA-MRSA. First, although ST97 *S. aureus* from human infections are often CA-MRSA, the emergence of FarR^H121Y^, FarR^C116Y^, and FarR^G166D^ in ST97 was restricted to bovine MSSA. Second, as previously noted for the FarR^H121Y^, C^116Y^, and E^160G^, we now find that FarR^G166D^ is also sufficient to confer enhanced resistance to linoleic acid among two distinct emergences of this variant in ST5 CA-MRSA. Third, we note the emergence of FarR^H121Y^ in USA300 strains from the UK, defined by an SCC*mec* VI element that encodes resistance to fusidic acid, and additional plasmid-mediated markers for resistance to mupirocin and quaternary amine disinfectants. Last, some ST228 strains of clonal complex 5 (CC5) HA-MRSA, which has become established in Europe, possess FarR^A14P^ with a substitution in the DNA-binding domain. Although this variant regulator is defective in DNA binding and unable to support FarE expression, these strains exhibit enhanced resistance to linoleic acid. Collectively, our data outline multiple pathways toward the evolution of both MSSA and MRSA with increased resistance to antimicrobial fatty acids that would be encountered at sites of colonization and infection.

## RESULTS

### ST228 strains with FarR^A14P^ exhibit increased resistance to linoleic acid independent of FarE

**Fig 1:**
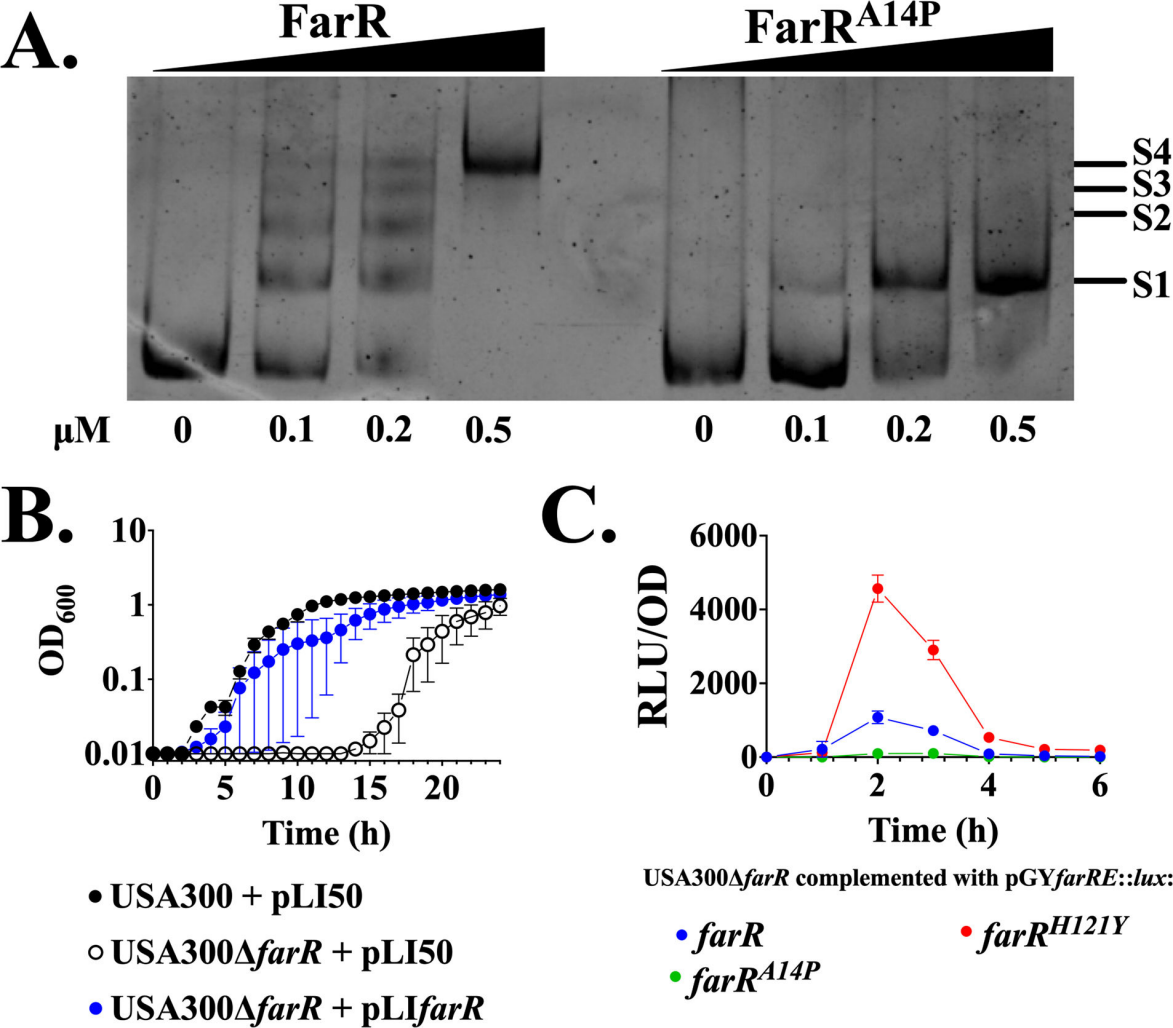
The FarR^A14P^ variant is sufficient to promote abrogated *farE* induction. (**A**) EMSA with 162 bp segment depicting the intergenic region between *farR* and *farE*. EMSA was conducted with 20 ng PCR probe and wild-type FarR protein or FarR^A14P^ as indicated. Protein-DNA complexes are labeled S1, S2, S3, and S4. Visualization was performed through ethidium bromide staining. (**B**) Cultures of USA300 or USA300Δ*farR* harboring pLI50 derivatives were inoculated to an optical density at 600 nm (OD_600_) = 0.01 into 96-well microtiter plates containing 200 µL of TSB supplemented with 50 µM linoleic acid + 0.1% dimethyl sulfoxide (DMSO), which is subinhibitory to wild-type USA300 during growth in microtiter plates. Plates were incubated at 37°C with orbital shaking, and growth (OD_600_) was monitored hourly. Each data point represents the mean ± standard error of the mean (SEM) from 6 × 200 µL wells in 96-well microtiter plates. (**C**) USA300Δ*farR* was transformed with either pGY*farRE::lux* or variant lux such as *farR*^H121Y^ or *farR*^A14P^, where luciferase activity is driven from the P*_farE_* promoter under the control of wild-type or variant farR genes. Cultures were grown in 125 mL flasks containing 25 mL TSB supplemented with 20 µM linoleic acid + 0.1% DMSO. Growth (OD_600_) and luciferase activity (relative light units [RLU]/OD_600_) were quantified at hourly intervals. This lower concentration of linoleic acid relative to panel B was chosen to accommodate growth of USA300Δ*farR*. Each data point represents the mean ± SEM from triplicate cultures.

FarR is required for expression of *farE* (13), and yet we previously noted a DNA-binding domain variant, FarR^A14P^ in the ST228 lineage of CC5 HA-MRSA (24), which potentially could abrogate its function as a regulator. Recent studies have described ST228 HA-MRSA as being endemic in Europe, including a phylogenetic analysis of 530 genomes from strains across 14 countries (29, 30). A search for FarR proteins that are identical to FarR^A14P^ identified 92 such strains (Table S1), most of which were sequenced in association with these studies. Since FarR^A14P^ is unique in having a non-conserved substitution in the N-terminal DNA-binding domain and this variant has documented persistence in Europe, we conducted experiments to assess the impact on FarR function, FarE expression, and resistance to antimicrobial fatty acids. First, we performed electrophoretic mobility shift assay (EMSA) with FarR or FarR^A14P^ and a probe comprised of the *farER* intergenic segment. Compared to FarR, which promotes four distinct shifts attributed to multiple operator sites (31), FarR^A14P^ displayed a single shift, indicative of diminished DNA binding (Fig. 1A). Since our previous work was conducted with a transposon insertion in *farR*, we constructed USA300Δ*farR* for precise evaluation of *farR* function. As expected, USA300Δ*farR* exhibited impaired growth in TSB + LA, which was restored with pLI*farR*, confirming that *farR* is required for resistance to linoleic acid (Fig. 1B). To assess expression, we constructed a *farRE::lux* reporter, where *farR* and the divergent *farE* promoter control luciferase expression. The *farRE::lux* construct promoted inducible *farE* expression in response to linoleic acid, while *farR*^H121Y^ promoted elevated expression as expected, and no expression was evident with *farR*^A14P^ (Fig. 1C).

**Fig 2:**
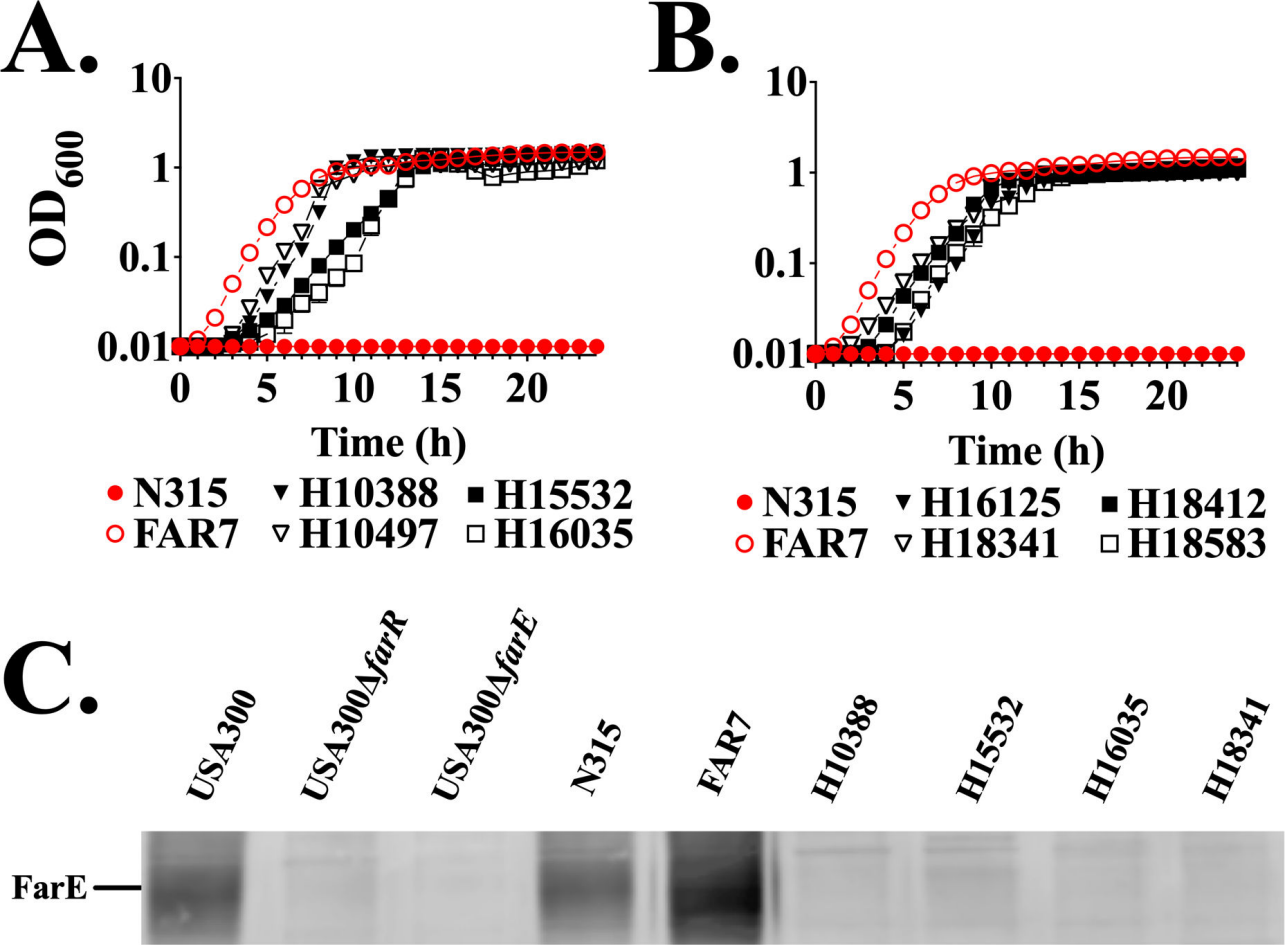
ST228 isolates harboring FarR^A14P^ display enhanced linoleic acid resistance independent of FarE. (**A and B**) Cultures of N315, FAR7, or MRSA harboring FarR^A14P^ from Switzerland were inoculated to an optical density at 600 nm (OD_600_) = 0.01 into 96-well microtiter plates containing 200 µL of TSB supplemented with 100 µM linoleic acid + 0.1% dimethyl sulfoxide. This concentration of linoleic acid restricts the growth of strains that do not have enhanced resistance phenotypes. Plates were incubated at 37°C with orbital shaking, and growth (OD_600_) was monitored hourly. Each data point represents the mean ± standard error of the mean from 6 × 200 µL wells in 96-well microtiter plates. (**C**) Western blot gels loaded with 3 µg of cell lysate protein from FarR^A14P^ variant strains. Gels were probed with FarE antisera. Protein concentration was normalized using the Bradford assay.

These data confirm that FarR^A14P^ cannot support FarE expression, and yet there are 92 *S*. *aureus* genomes with this variant (Table S1), primarily from Europe, with SCC*mec* 1B and *spa* type t1003. We obtained eight such strains (Table S2) associated with an MRSA outbreak in Swiss hospitals (30) and assayed their growth in TSB + 100 µM linoleic acid relative to *S. aureus* N315 and the *in vitro*-selected FAR7 strain, which expresses FarR^H121Y^. As expected, ST5 HA-MRSA strain N315 did not grow under this restrictive condition, while FAR7 exhibited unimpeded growth, but surprisingly, all ST228 FarR^A14P^ strains also grew effectively (Fig. 2A and B), and all but one of these (strain 16035) exhibited an MIC for linoleic acid in excess of 800 µM, exceeding our previously determined MIC of *S. aureus* USA300 CA-MRSA (400 µM) and the ST5 HA-MRSA reference strain N315 (Fig. S1). A Western blot assay confirmed that the enhanced growth of ST228 MRSA relative to strain N315 could not be attributed to FarE, since N315 HA-MRSA exhibited FarE expression comparable to USA300 CA-MRSA, whereas ST228 HA-MRSA exhibited negligible FarE expression comparable to USA300Δ*farR* (Fig. 2C; Table S3), consistent with these strains having a non-functional FarR regulator. From these data, we conclude that, in the absence of a functional FarR regulator, ST228 MRSA have evolved compensatory mechanisms to achieve enhanced resistance to antimicrobial fatty acids.

### Emergence of ST5 strains with FarR^G166D^ and sufficiency to confer increased resistance to linoleic acid

**Fig 3:**
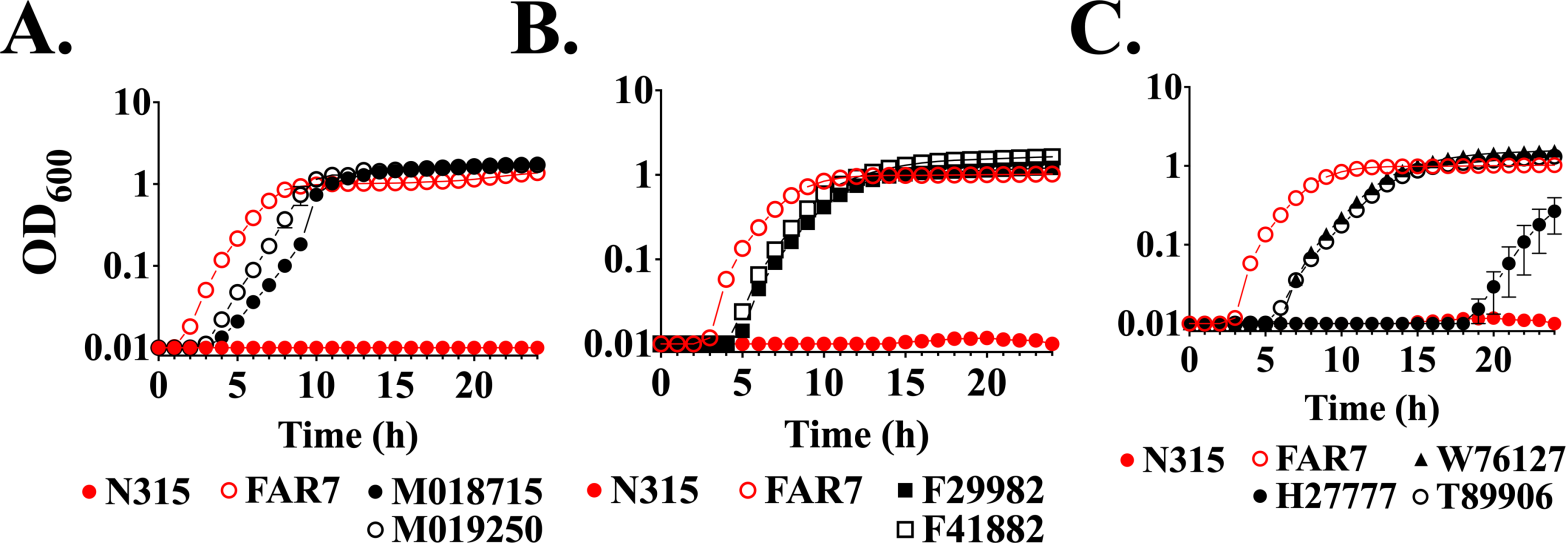
ST5 isolates harboring FarR^G166D^ display enhanced linoleic acid resistance. Cultures were inoculated to an optical density at 600 nm ( OD_600_) = 0.01 into 96-well microtiter plates containing 200 µL of TSB supplemented with a subinhibitory concentration of 100 µM linoleic acid + 0.1% dimethyl sulfoxide. This concentration of linoleic acid restricts growth of strains that do not have enhanced resistance phenotypes. Plates were incubated at 37°C with orbital shaking, and growth (OD_600_) was monitored hourly. (**A**) FarR^G166D^ strains from the Netherlands, and (**B and C**) FarR^G166D^ strains from the United States. Each data point represents the mean ± standard error of the mean from 6 × 200 µL wells in 96-well microtiter plates.

We previously noted the polyphyletic emergence of ST5 MRSA with FarR^G166D^ in North America and Europe (24). A list of 27 *S*. *aureus* genomes with FarR^G166D^ identified using the Identical Protein function in the NCBI database of *S. aureus* subsp. *aureus* proteins is provided in Table S4, and a subset of these, including ST5 CA-MRSA obtained for this study, is detailed in Table S2 with metadata on accession and NCBI BioSample numbers, common strain name, country of origin, *spa* type, MLST, SCC*mec,* and antimicrobial resistance genes. Surprisingly, many of these were MSSA, including ST8 from Japan and the United States, ST672 from Japan, and ST97 from New Zealand. MRSA with FarR^G166D^ was predominantly ST5 or ST8 CA-MRSA harboring SCC*mec* IV or V, respectively (Table S2). We acquired two MRSA with FarR^G166D^ and SCC*mec* V from Denmark, and five from North America with SCC*mec* IV (Table S2), representing the two emergences of this variant among ST5 MRSA. Most of these strains initiated growth after a lag phase of 3–5 h in TSB + 100 µM LA, whereas *S. aureus* N315 exhibited no growth after 24 h, and FAR7 exhibited enhanced growth relative to strains with FarR^G166D^ (Fig. 3A through C). In a Western blot assay that assessed FarE production in CC5 CA-MRSA representing the separate emergences of FarR^G166D^ in the United States and the Netherlands, FarE expression was variable. Strains H27777 from the United States and M01875 from the Netherlands exhibited elevated FarE relative to the *S. aureus* N315 ST5 HA-MRSA reference strain, but not to the same extent as FAR7, which expresses FarR^H121Y^. Alternatively, expression of FarE in strains T89906 and M019250 from the United States and the Netherlands, respectively, was comparable to N315 (Fig. S2A, Table S5). Despite the variable expression of FarE, all strains exhibited an MIC > 800 µM linoleic acid (Fig. S1B and C).

**Fig 4:**
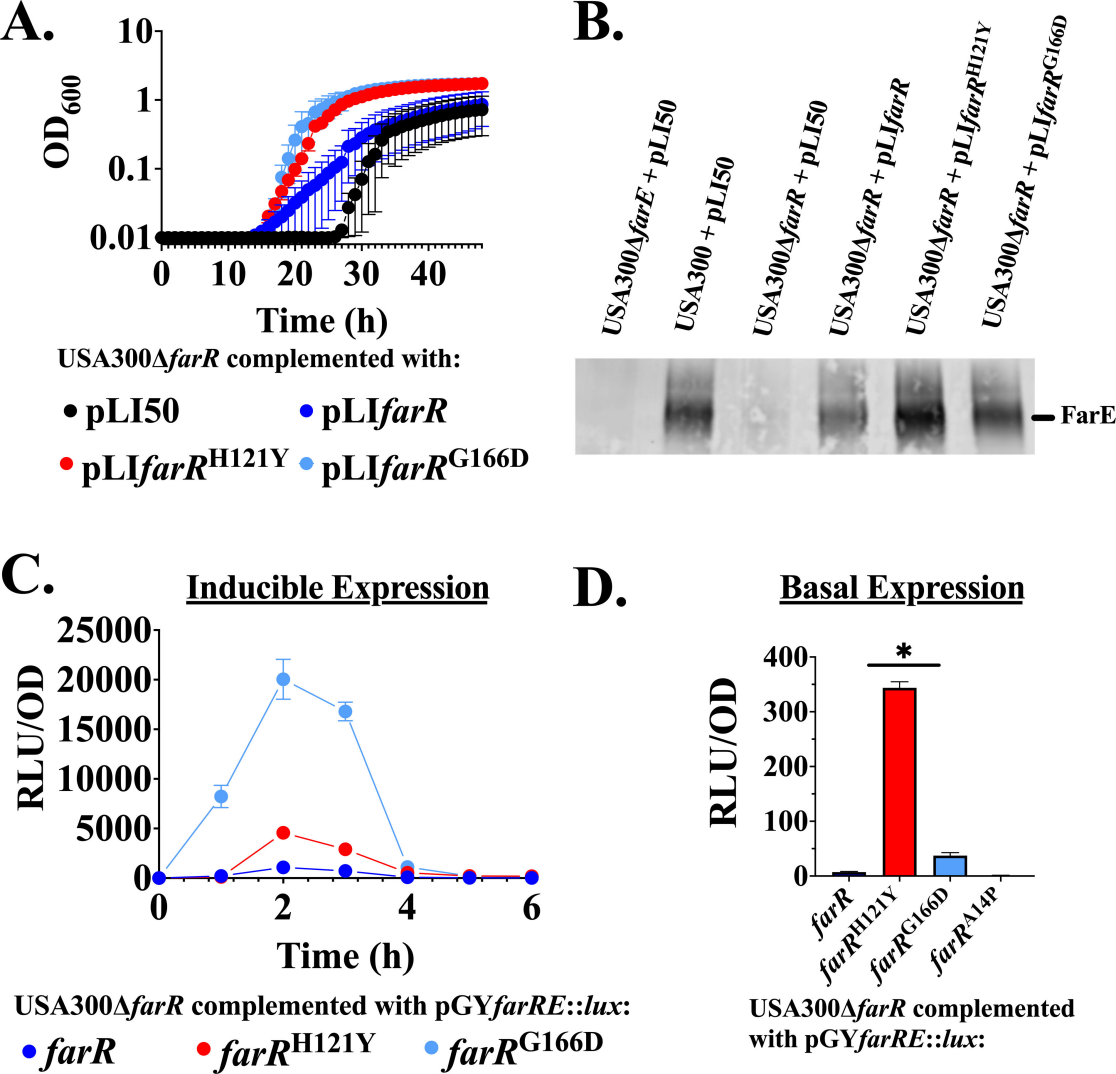
The FarR^G166D^ variant is sufficient to promote increased linoleic acid resistance. (**A**) Cultures of USA300Δ*farR* transformed with pLI50 vehicle or derivatives harboring FarR^G166D^ were inoculated to an optical density at 600 nm (OD_600_) = 0.01 into 96-well microtiter plates containing 200 µL of TSB supplemented with 100 µM linoleic acid + 0.1% dimethyl sulfoxide (DMSO), which restricts growth of strains that do not exhibit enhanced resistance phenotypes. Plates were incubated at 37°C with orbital shaking, and growth (OD_600_) was monitored hourly. Each data point represents the mean ± standard error of the mean (SEM) from 6 × 200 µL wells in 96-well microtiter plates. (**B**) Western blot gels were loaded with 5 µg of cell lysate protein from USA300, USA300Δ*farE,* and USA300Δ*farR* harboring pLI50 and derivatives of FarR. Blots were probed with FarE antisera. Protein concentration was normalized using Bradford assay. (**C**) USA300Δ*farR* was transformed with either pGY*farRE::lux* or variant lux such as farR^H121Y^ and farR^G166D^, where luciferase activity is driven from the P*_farE_* promoter under the control of wild-type or variant farR genes. Cultures were grown in 125 mL flasks containing 25 mL TSB supplemented with 20 µM linoleic acid + 0.1% DMSO. This lower concentration of linoleic acid relative to panel A was chosen to permit growth of USA300Δ*farR* harboring the wild-type *farR* reporter, and also reflects increased sensitivity of bacteria to linoleic acid in flask cultures compared to growth in microtiter plates. Growth (OD_600_) and luciferase activity (relative light units [RLU]/OD_600_) were quantified at hourly intervals. (**D**) To determine basal expression, pGY*farRE::lux* or variant lux cultures were grown in 125 mL flasks containing 25 mL of TSB supplemented with 20 µM linoleic acid + 0.1% DMSO. Growth (OD_600_) and luciferase activity (RLU/OD_600_) were quantified during stationary phase. Each data point represents the mean ± SEM from triplicate cultures. Statistically significant differences (*, *P* < 0.05) compared to *farR* were determined by Tukey’s multiple-comparison test.

In view of these variable outcomes, we conducted additional work to assess the sufficiency of FarR^G166D^ for enhanced resistance to linoleic acid by complementing USA300Δ*farR* with either pLI*farR*, *farR*^H121Y^, or *farR*^G166D^. The pLI*farR*^H121Y^ and pLI*farR*^G166D^ constructs both promoted enhanced growth of USA300Δ*farR* relative to wild-type pLI*farR* in TSB + 100 µM linoleic acid (Fig. 4A). When these same strains were tested for expression of FarE in a Western blot assay, complementation with pLI*farR*^H121Y^ promoted elevated FarE production, whereas partially elevated expression was evident with pLI*farR*^G166D^ (Fig. 4B; Table S6). To further assess the impact of farR^G166D^ on FarE expression, we constructed *farRE::lux* reporters where wild-type or variant *farR* alleles and the adjacent *farE* promoter segment drive luciferase expression. Strikingly, *farR*^H121Y^ and *farR*^G166D^ both promoted elevated inducible and basal expression of *farE* relative to native *farR* (Fig. 4C and D), and surprisingly, the *farRE::lux* construct with *farR*^G166D^ promoted far higher expression compared to *farR*^H121Y^. From this analysis, it is apparent that ST5 CA-MRSA with *farR*^G166D^ has exhibited two distinct emergences in the phylogenetic structure of ST5 HA-MRSA. Although these strains exhibit enhanced growth in TSB with 100 µM linoleic acid relative to ST5 HA-MRSA strain N315, elevated FarE production was not consistent across these strains. Moreover, whereas *farR*^H121Y^ consistently confers elevated *farE* transcription and enhanced production of FarE protein, *farR*^G166D^ promoted strongly elevated transcription of *farE* that did not correlate with a proportional increase in FarE protein relative to FarR^H121Y^. This alludes to an intricate relationship between *farE* polymorphisms, regulatory function, and production of FarE protein, which may also be modulated through post-transcriptional processes and accrual of additional polymorphisms in different clinical isolates with FarR^G166D^.

### Multiple emergences of variant FarR regulators across ST97 bovine MSSA

**Fig 5:**
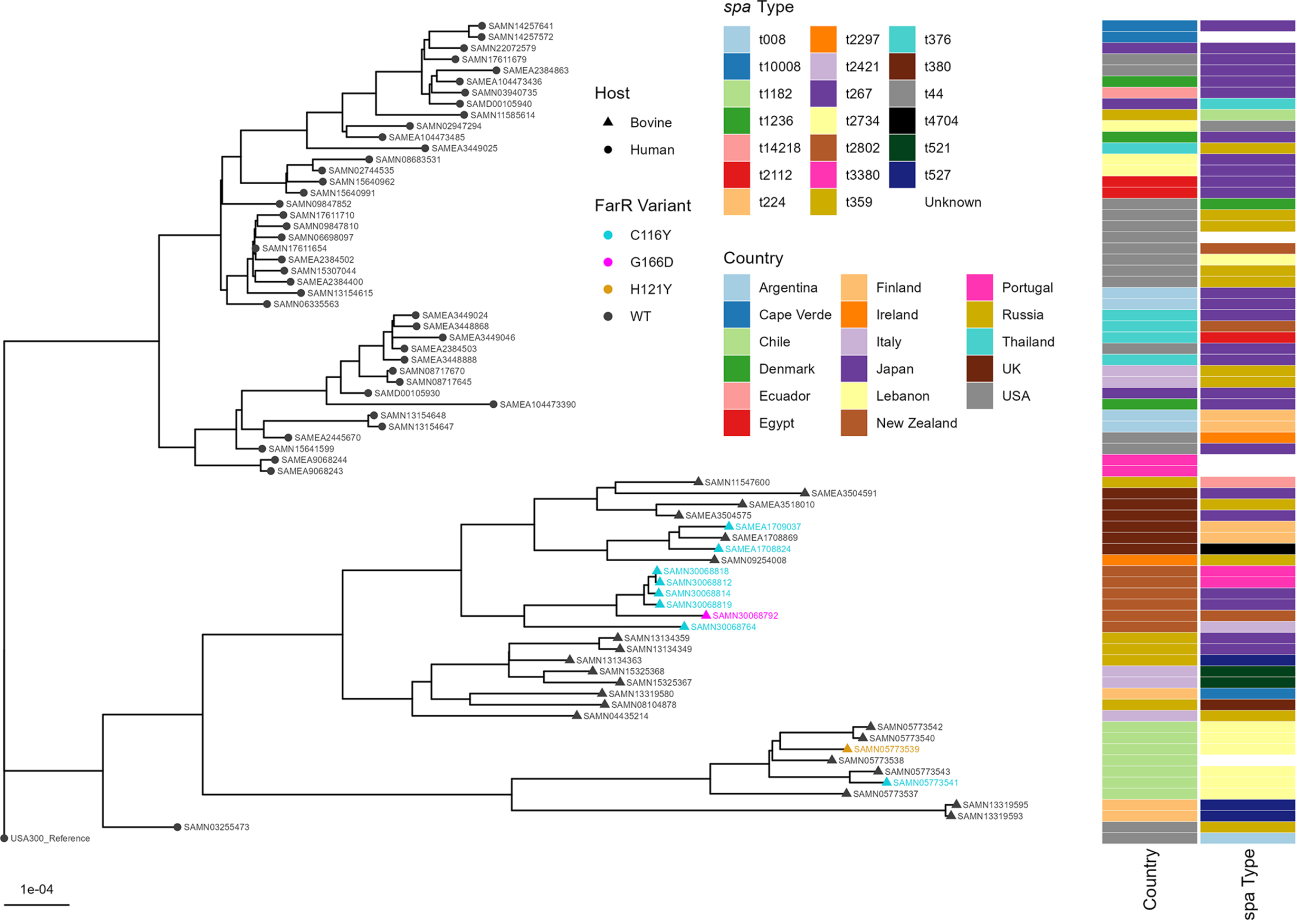
Distribution of *farR* variants across the phylogenetic spectrum of *S. aureus* ST97. Reads from all *S. aureus* genome sequences were mapped to the *S. aureus* USA300_FPR3757 reference genome (GCF_000013465.1) using Snippy-core (v.4.6.0) (33). A core SNP alignment was generated using Snippy-core and used to construct a maximum-likelihood phylogeny with IQ-TREE (v.2.2.2.7) (34). The tree was rooted using the *S. aureus* USA300_FPR3757 genome as an outgroup. Here, strains with variant FarR proteins are placed in the context of ST97 phylogeny. Biosample numbers from the NCBI genome sequence entries for each strain are shown adjacent to the branch structures. These strains are listed in the same order in Table S7, which also provides their Pathosystems Resource Integration Center accession numbers (where available) or common strain name, country of origin, host background, and *spa* type. Columns adjacent to the Biosample numbers provide information on country of origin (Country) and *spa* type, while tip shape is indicative of host background. FarR variant types are indicated by the color of the BioSample number.

We previously reported on multiple variant FarR regulators in CC5 MRSA, of which FarR^C116Y^ and FarR^G166D^ also occurred in CC97. CC5 and CC97 were also remarkable in having a higher proportion of strains with variant FarR compared to other major clonal complexes (24). Within CC5, such strains had emerged multiple times across the phylogenetic structure. We therefore conducted a similar analysis to assess the emergence of variant FarR regulators across the phylogenetic structure of ST97. For this, we used genomes from a cross-section of 74 human and bovine strains, including those with FarR variants, that were designated complete in the BV-BRC Bacterial and Viral Informatics Resource Center (https://www.bv-brc.org/) (32). Metadata for these strains appears in Table S7, and a summary of all FarR variants identified within CC97 is presented in Table 1. Human and bovine ST97 were phylogenetically distinct (Fig. 5), and among bovine isolates, several strains from Chile, Finland, and the United States were phylogenetically distinct from a larger branch comprised of strains from Russia, Italy, the UK, Ireland, and New Zealand. Strains from Chile included FarR^C116Y^ and FarR^H121Y^, both with *spa* t2734 that emerged from a common ancestor, while strains from New Zealand and the UK with FarR^C116Y^ that were mostly *spa* t224, t3380, and t267 also evolved from a common branch point. Among this cluster was also a single *spa* t2802 strain with FarR^G166D^, which appears to represent another example of different FarR variants having emerged from a common branch. Of note, FarR^C116Y^ was previously shown to have emerged among CC5 HA-MRSA and displayed sufficiency for increased linoleic acid resistance dependent on FarE (24). Therefore, as previously noted with CC5 MRSA, there have been multiple geographic emergences of FarR variants among ST97 bovine MSSA, including geographic dispersal of a cluster of related strains with FarR^C116Y^ across the UK and New Zealand. In addition to these two countries, other CC97 strains with FarR^C116Y^ were found in Ireland, Switzerland, Chile, and China (Table 1).

**TABLE 1:** Strains with variant FarR regulators in CC97

Variant (*n*)^*a*^	Country	MLST	Spa type^*b*^
FarR^H121Y^ (2)	UK, Chile	1859, ST97	New, t2734
FarR^G166D^ (4)	UK, New Zealand	97, 97, 3087, 3112	t267, t524, t2802, t9417
FarR^C116Y^ (23)	UK, Ireland, Switzerland, Chile, New Zealand, China	97 (*n* = 16), 71 (*n* = 3), 3109, 3221, 3173, New	t224 (*n* = 4), t267 (*n* = 4), t359, t521, t524 (*n* = 3), t693, t1190 (*n* = 2), t1418, t2421, t2734, t3380 (*n* = 2), t6297

^*a*^ Obtained through BLAST homology searches for each single amino acid substitution variant, combined with use of the Identical Proteins link at NCBI.

^*b*^ Underlined *spa* types represent where different variants have emerged in common spa types.

### ST8 CA-MRSA with FarR^H121Y^ exhibit increased resistance to linoleic acid

**Fig 6:**
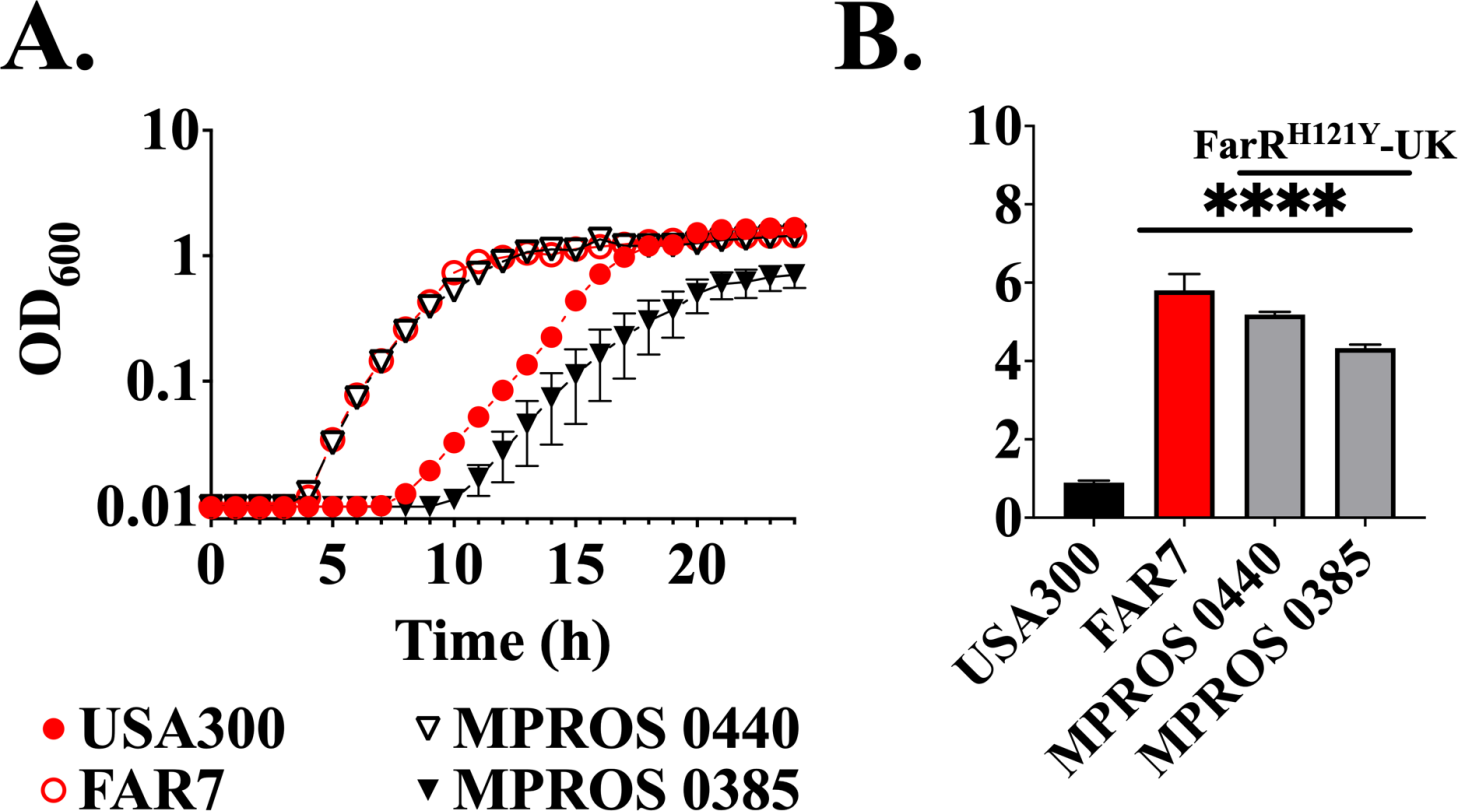
ST8 strains harboring FarR^H121Y^ promote enhanced antimicrobial resistance. (**A**) Cultures were inoculated to an optical density at 600 nm (OD_600_) = 0.01 into 96-well microtiter plates containing 200 µL of TSB supplemented with 100 µM linoleic acid + 0.1% dimethyl sulfoxide (DMSO), which is sufficient to restrict the growth of strains that do not exhibit an enhanced resistance phenotype. Plates were incubated at 37°C with orbital shaking, and growth (OD_600_) was monitored hourly. Each data point represents the mean ± standard error of the mean (SEM) from 6 × 200 µL wells in 96-well microtiter plates. (**B**) Cultures of USA300, FAR7, or MRSA harboring FarR^H121Y^ from the UK were inoculated to OD_600_ = 0.01 into triplicate tubes containing 3 mL of TSB supplemented with 800 µM linoleic acid + 0.1% DMSO, which is 2× MIC previously reported for wild-type USA300 (31). Tubes were incubated at 37°C with orbital shaking, and growth (OD_600_) was determined after 24 h. Each data point represents the mean ± SEM from triplicate cultures. Statistically significant differences (***, *P* < 0.001; *, *P* < 0.05) compared to USA300 or N315 were determined by Tukey’s multiple-comparison test.

Our identification of *farER* was facilitated through *in vitro* selection for increased resistance to linoleic acid in the USA300 strain of CA-MRSA, leading to recovery of strain FAR7 with FarR^H121Y^. Although this variant was not evident in ST5 USA100 HA-MRSA, in which we had noted the emergence of several other FarR variants, we did note the sporadic emergence of FarR^H121Y^ in different clonal complexes, which in addition to CC97 included CC1, CC8, CC22, and CC30 (Table S2). With the availability of more genome sequences, we now note the emergence of FarR^H121Y^ in eight strains defined as ST8 spa t190 CA-MRSA, possessing an SCC*mec* VI element harboring *fusC* for fusidic acid resistance (Table S2). These CA-MRSA from the UK had additional resistance genes *ermA, ant(9)-Ia, tetM, mupA*, and QacA for resistance to erythromycin, aminoglycoside, tetracycline, mupirocin, and quaternary amine disinfectant, respectively (Table S2). We obtained two such strains with FarR^H121Y^ from the UK and assessed their growth in TSB containing 100 µM linoleic acid, where FAR7 and MPROS 0440 initiated exponential growth after a 4 h lag, while USA300 and MPROS 0385 initiated growth after a lag of 8–10 h (Fig. 6A). To confirm elevated resistance in these strains, endpoint growth was assessed after 24 h in TSB with 800 µM LA. As expected, FAR7 exhibited strong growth after 24 h, and both ST8 CA-MRSA clinical isolates with FarR^H121Y^ exhibited growth comparable to FAR7 (Fig. 6B). Therefore, although MPROS 0440 alone displayed enhanced growth in TSB + 100 µM LA, both MPROS 0440 and MPROS 0385 exhibited an MIC >800 µM. A Western blot assay of FarE expression in these ST8 spa t190 clinical isolates compared to the *in vitro*-selected FarR^H121Y^ variant strain FAR7, together with ST5 HA-MRSA strain N315, is presented in Fig. S2B and Table S8. Strain FAR7 and the two ST8 *spa* t190 CA-MRSA both exhibited elevated FarE production relative to the USA300 and N315 strains harboring wild-type FarR, indicating that the emergence of FarR^H121Y^ in ST8 spa t190 CA-MRSA promotes an enhanced resistance to linoleic acid through increased expression of FarE.

## DISCUSSION

Our identification of FarE and its divergently transcribed TFR FarR in USA300 occurred through *in vitro* selection for increased resistance to linoleic acid in the widely disseminated USA300 strain of CA-MRSA. FarR harbored an H^121^Y substitution which conferred increased resistance to skin-relevant antimicrobial fatty acids and increased expression of *farE* (13). In related work, variants in FarR and an orthologous TetR family regulator in *Staphylococcus epidermidis* were recovered in *S. aureus* and *S. epidermidis* strains that were subjected to *in vitro* selection for enhanced resistance to sphingosine, a monounsaturated 18-carbon amino alcohol with antimicrobial activity, that is derived from hydrolysis of epidermal ceramides (35). Given that enhanced resistance to antimicrobial lipids present on skin was readily achieved through *in vitro* selection, we hypothesized that *in vivo* exposure to host-derived fatty acids through repeated cycles of colonization, infection, and transmission among MRSA in a hospital setting should also promote the emergence of MRSA with increased resistance to antimicrobial fatty acids. This was supported through our identification of FarR variants among ST5 MRSA, including polyphyletic emergences of FarR^C116Y^ and FarR^G166D^ (24), and we demonstrated that FarR^C116Y^ was sufficient to promote enhanced resistance to linoleic acid in this genetic background. Our present work extends these findings, revealing that evolution of enhanced resistance to antimicrobial uFFA through multiple emergences of variant FarR regulators is not restricted to ST5 HA-MRSA. First, we have confirmed that FarR^G166D^ is sufficient to confer increased resistance to linoleic acid in ST5 CA-MRSA. Second, we have established that CC97 MSSA of bovine origin represents another genetic background with multiple emergences of variant regulators FarR^G166D^, FarR^C116Y^, and FarR^H121Y^ that promote enhanced resistance to antimicrobial uFFA. Third, while we previously noted the sporadic occurrence of FarR^H121Y^ in different genetic backgrounds, we have now documented the emergence of this variant that confers high resistance to antimicrobial uFFA in USA300 CA-MRSA from the UK.

It is noteworthy that FarR^C116Y^ and FarR^H121Y^ have emerged in multiple different genetic backgrounds, while *in vitro* selection for enhanced resistance to sphingosine, another antimicrobial lipid relevant to skin colonization, led to recovery of FarR^D94Y^ in two *S*. *aureus* variants with enhanced resistance, and an F^110^Y substitution in an orthologous regulator in two *S*. *epidermidis* variants with enhanced resistance (35). The affinity of TFRs for their target regulatory motifs is typically modulated through binding of small molecules, and recent studies have shown the importance of free tyrosine residues in mediating ligand binding within multidrug resistance (MDR) regulators. For example, disruption of hydrogen bonding due to an E253A substitution frees Y152 for ligand binding in the BmrR regulator of MDR transporters from *Bacillus subtilis* (36). A similar mechanism is observed in QacR of *S. aureus,* where Y103 and Y123 are highly flexible to better accommodate ligand binding (37). Although the ligand-binding mechanism of FarR remains to be determined, a potential means by which FarR^H121Y^ and FarR^C116Y^ may upregulate *farE* expression is through substitution with flexible tyrosine residues that enhance ligand binding, and similar considerations could apply to D^94^Y and F^110^Y variants of orthologous FarR regulators identified through *in vitro* selection for enhanced resistance to sphingosine in *S. aureus* and *S. epidermidis*, respectively (35).

Considering that *in vitro* exposure of USA300 to growth-inhibitory linoleic acid led to our discovery of FarR^H121Y^ (13), an emergence of this variant within USA300 clinical isolates was not unexpected. While USA300 CA-MRSA in North America are typically ST8 spa t008 with SCC*mec* type IV (38), those with FarR^H121Y^ in our present study were identified in the UK and were ST8 *spa* t190 with a novel SCC*mec* type VI harboring *fusC* (39). MRSA first emerged in the UK as ST8 strains designated EMRSA-2 and EMRSA-6 with *spa* t190 and SCC*mec* type IV (40). Subsequently, *spa* t190 MRSA was classified as a nosocomial lineage of ST8 HA-MRSA harboring SCC*mec* II (41). Therefore, it appears that within the UK, an ancestral ST8 *spa* t190 strain has at different times acquired SCC*mec* IV, II, and VI. Previously, SCC*mec* VI has been predominantly characterized among CC30 strains, with sporadic instances among CC8 and CC5 (41, 42). Our description of FarR^H121Y^ in ST8 CA-MRSA with SCC*mec* VI is consistent with evolution toward enhanced colonization through increased resistance to unsaturated fatty acids and topical antimicrobial agents used to treat superficial infections and/or in decolonization therapy.

Moreover, although USA300 CA-MRSA is typically not resistant to multiple antimicrobial agents, ST8 *spa* t190 SCC*mec* VI MRSA with FarR^H121Y^ has an MDR phenotype with *fusC, ermA, ermC, tetM, ant(9)-Ia,* and *mupA,* alongside quaternary amine and biguanide resistance. The *fusC* gene on SCC*mec* VI confers resistance to fusidic acid, which is often used as a topical treatment for superficial skin and ocular infections, while *mupA* encodes an alternative isoleucyl-tRNA synthase (*ileS2*) that confers high-level resistance to mupirocin, which is used for decolonization of *S. aureus* nasal carriage (43, 44). These strains also carried *qacA* for resistance to quaternary amines and biguanides, which are heavily used as topical and surface disinfectants in a nosocomial setting. Strikingly, MPROS0440 also possessed a premature stop codon in the QacR TFR responsible for repression of QacA (45, 46), such that the QacA efflux pump should be de-repressed and confer elevated resistance to quaternary amines. Finally, the *ermA, ermC, tetM,* and *ant(9)-Ia* genes are typically not co-associated with USA300 CA-MRSA but are common in MDR HA-MRSA, conferring resistance to macrolides, tetracycline, and spectinomycin, respectively. Cumulatively, these observations point toward a CA-MRSA strain evolving to achieve enhanced colonization of humans through increased resistance to antimicrobial fatty acids and topical antimicrobials employed to treat superficial skin infections, while bridging the gap between CA-MRSA and HA-MRSA through an MDR phenotype.

Just as our previous work identified ST5 USA100 HA-MRSA as a genetic background that supports multiple emergences of variant FarR regulators, we now find that this also occurs in CC97 *S. aureus* with FarR^C116Y^, FarR^G166D^, and FarR^H121Y^. Historically, ST97 *S. aureus* have been strongly associated with porcine and bovine hosts and appear to harbor more drug resistance than other animal isolates (28, 47). However, ST97 strains have also made the jump to human hosts with a diverse array of MDR infections around the world (25–27). Our analysis has confirmed that human and bovine CC97 *S. aureus* are phylogenetically distinct. Unexpectedly, although an MDR phenotype is well documented among both human and animal CC97 *S. aureus*, our analysis revealed that CC97 strains with variant FarR regulators were exclusively MSSA of bovine origin, including a cluster of related isolates with FarR^C116Y^ from the UK and New Zealand, documenting the geographic spread of this variant. Given the significant association of *S. aureus* with bovine mastitis (28, 48), the lipid-rich composition of milk combined with farming practices could contribute to the emergence and dissemination of this strain. Notably, the cis-uFFAs oleic acid and linoleic acid, as well as the ruminant-specific trans-uFFA rumenic acid, are significant components of lipid-rich milk (49), and the practice of multiple daily automated milking also promotes a significant increase in the free fatty acid content of milk (50, 51).

These data support a potential role for positive selection in association with dairy practices in contributing to the emergence of *S. aureus* strains with enhanced resistance to antimicrobial uFFA. Thus, while our original interpretation of multiple emergences of variant FarR regulators within ST5 USA100 HA-MRSA led us to hypothesize that this was due to positive selection imposed through repeated cycles of colonization, infection, and transmission in a nosocomial setting, our present findings establish that this can also occur in the context of MSSA. Indeed, although we first reported two independent emergences of FarR^G166D^ among ST5 CA-MRSA, our present analysis notes the emergence of this variant among MSSA of diverse MLSTs, which, in addition to bovine CC97, also includes ST8, ST1004, ST15, and four ST672 strains from Japan. The ST672 MSSA strains had *spa* types t5156 and t1309, of which the latter has been associated with a highly virulent and newly emergent ST672 CA-MRSA in India (52). However, the extent to which this and other FarR variants are established among MSSA cannot be accurately assessed, since, with the exception of mastitis in dairy cattle, MSSA are less likely to be subject to surveillance and genome sequencing projects compared to MRSA.

Although our focus has been on the emergence of variant FarR regulators that promote increased resistance to antimicrobial uFFA due to elevated expression of FarE, we must also consider potential fitness costs. The AlphaFold structure of FarE reveals a strong similarity to MmpL3 of *Mycobacterium tuberculosis,* which has a critical role in the transport of a lipid precursor required for the synthesis of the essential cell wall component, mycolic acid (53). While we first reported that FarE promoted the efflux of linoleic acid, others described a FarR^C116R^ variant that promotes overexpression of FarE concomitant with accumulation of membrane vesicles in culture supernatant, such that a role in phospholipid efflux was proposed (54, 55). Strikingly, while our analysis of ~40,000 publicly accessible *S. aureus* genomes identified variant regulators FarR^C116Y^, FarR^H121Y^, and FarR^G166D^ across a phylogenetically diverse spectrum of *S. aureus* isolates from human and bovine hosts, FarR^C116R^ has thus far only been identified through *in vitro* selection, which may reflect a strong counterselection against the emergence of this variant in human and animal hosts. In this respect, our data revealed that a *farRE::lux* reporter constructed with FarR^H121Y^ promoted elevated expression of *farRE::lux* as expected, but, surprisingly, this level of expression was exceeded with FarR^G166D^. Nevertheless, the amount of FarE produced by ST5 CA-MRSA with FarR^G166D^ was comparable to, but did not exceed, that produced by MRSA that express FarR^H121Y^. Moreover, production of FarE among ST5 CA-MRSA with FarR^G166D^ was variable, with some strains being comparable to the *in vitro*-selected FarR^H121Y^ FAR7 strain, while others were similar to the ST5 HA-MRSA reference strain N315. Although speculative, in consideration of the ability of FarE to promote phospholipid efflux (54), it may be that prolonged high expression of FarE is detrimental to viability. Indeed, studies have shown that under certain stress conditions, limiting phospholipid efflux confers an advantage to gram-negative bacteria through the preservation of outer membrane asymmetry and integrity (56). Potentially, the transmission of strains with variant FarR regulators is limited by a fitness cost attributed to elevated FarE expression, which could then select for compensatory mutations. These considerations may account for the variable expression of FarE we have noted in strains with FarR^G166D^.

Considering the potential deleterious consequences associated with elevated FarE expression, it may be advantageous for *S. aureus* to achieve increased resistance to antimicrobial uFFA through mechanisms that are independent of FarE. One such example is evident with FarR^E93EE^, in which there has been a duplication of a codon for glutamic acid at position 93 in the FarR coding sequence. This variant emerged among ST5 HA-MRSA that exhibited increased resistance to linoleic acid, even though FarR^E93EE^ alone was not sufficient to confer that phenotype (24). While this variant first appeared in ST5 HA-MRSA, our phylogenetic analysis of strains with FarR^E93EE^ noted that this variant within ST5 then evolved to ST764, which is a single-locus MLST variant of ST5, concomitant with the accumulation of several pseudogenes compared to the parent ST5. These ST764 strains with FarR^E93EE^ were *spa* t1084 (24), and ST764 *spa* t1084 HA-MRSA has been noted as an emerging hypervirulent HA-MRSA clone in China (57). In the NCBI database, there are currently 868 protein accessions for Far^E93EE^, many of which are from genomes that were sequenced to describe the emergence of this ST764 spa t1084 HA-MRSA clone (57).

Another example of evolutionary pressure being exerted by, or concomitant with, a variant FarR regulator is evident in the FarR^A14P^ isolates described in our present study, which were more resistant to linoleic acid even though FarR^A14P^ was deficient in DNA binding and unable to support inducible expression of FarE. These HA-MRSA, primarily from Swiss hospitals, were ST228, which is a double-locus MLST variant of ST5, and exhibited enhanced resistance to linoleic acid even though inducible expression of FarE was not evident. One possible explanation for the maintenance of resistance in the absence of FarE is the accumulation of pseudogenes, which may contribute to altered phenotypic traits through mechanisms that are challenging to predict, as previously noted with ST764 strains that express FarR^E93EE^ (24). Overall, the documented success of ST228 HA-MRSA with FarR^A14P^ suggests that these strains tolerate impaired *farE* induction, potentially due to compensatory mechanisms such as pseudogene-associated adaptations. Alternatively or additionally, as noted in a previous study that employed a systemic approach to address mechanisms of resistance to unsaturated long-chain fatty acids, multiple contributory genes were identified, including metabolic, regulatory, efflux, and cell envelope-related functions (58). Therefore, a more detailed analysis of strains that exhibit enhanced resistance to antimicrobial fatty acids through *farER*-independent mechanisms is warranted.

In conclusion, we have defined multiple emergences of *S. aureus* clinical isolates with variant FarR regulators and enhanced resistance to antimicrobial fatty acids across a diverse spectrum of genetic backgrounds and antimicrobial susceptibilities. Of these variant FarR regulators, FarR^H121Y^, FarR^C116Y^ and FarR^G166D^, and FarR^E160G^ are sufficient to promote enhanced resistance to linoleic acid, and one factor that may limit the rampant dissemination of these strains could be the deleterious consequences of elevated phospholipid efflux. Of concern, we have noted the emergence of FarR^H121Y^ in an ST8 CA-MRSA background that bridges the distinctions between HA-MRSA and CA-MRSA by exhibiting an MDR phenotype that includes enhanced resistance to antimicrobial unsaturated fatty acids that would be encountered on skin, as well as fusidic acid and mupirocin, which are used for decolonization therapy and to treat superficial skin infections. Conversely, the emergence of FarR^E93EE^ and FarR^A14P^ represents substitutions that are not sufficient to promote enhanced resistance, and yet *S. aureus* strains with these variant regulators appear to have evolved enhanced resistance to antimicrobial uFFA through FarR-independent mechanisms that avoid any deleterious consequences of elevated efflux pump expression. While our findings highlight the potential impact of FarER variation on uFFA resistance, it is likely that enhanced resistance across diverse genetic backgrounds reflects the influence of multiple loci, as first identified in a systematic screen for genetic contributors to linoleic acid resistance (58). As genome sequencing becomes a mainstream tool for surveillance and monitoring of MDR phenotypes, the future will reveal to what extent these strains with enhanced resistance to antimicrobial uFFA become established within HA- and CA-MRSA, as well as MSSA strains.

## MATERIALS AND METHODS

### Cultures, plasmids, and growth conditions

*S. aureus* and *Escherichia coli* strains and plasmids that were constructed for this study, and variant FarR strains obtained for this study, are listed in Table 2. *S. aureus* clinical isolates with variant FarR proteins (FarR^G166D^, FarR^A14P^, FarR^H121Y^) were obtained from collections maintained by the Massachusetts Microbiome Center, Sandra Witteveen from the National Institute for Public Health and the Environment (Netherlands), Dominique Blanc from the Lausanne University Hospital, and Sharon Peacock at the University of Cambridge. *S. aureus* and *E. coli* were cultured in TSB and LB, respectively, containing 15 g/L agar when required for growth on solid medium. Cultures were maintained at −80°C in 20% glycerol; when required for experimental purposes, cultures from frozen stocks were streaked on agar medium, and single colonies were inoculated into polypropylene tubes containing 3 mL of the appropriate broth and grown overnight at 37°C on an orbital shaker at 220 rpm. When necessary for plasmid maintenance, TSB was supplemented with 5 µg/mL chloramphenicol, and LB was supplemented with 100 µg/mL ampicillin.

**TABLE 2:** *S. aureus* and *E. coli* strains and plasmids

Strain or plasmid	Description^*a*^	Reference or Biosample ID
*S. aureus*		
RN4220	Restriction endonuclease deficient strain capable of accepting foreign DNA (rk– mk+)	(59)
USA300	CA-MRSA, wild-type strain cured of resistance plasmids	(32, 60)
N315	ST5 HA-MRSA	(61)
FAR7	*In vitro*-selected FarRH121Y variant of USA300; increased resistance to linoleic acid	(13)
USA300 pKOR*farR*	USA300 with pKOR*farR* shuttle vector for markerless *farR* deletion; Cm^r^	This study
USA300Δ*farR*	USA300 with markerless *farR* deletion	This study
USA300Δ*farR* pLI50	USA300Δ*farR* with pLI50 complementation vector; Cm^r^	This study
USA300Δ*farR* pLI*farR*^xy^	USA300Δ*farR* complemented with *farR* cloned in pLI50; *farR^xy^* segment derived from USA300, FAR7 (*farR^H121Y^*), or SDM of pLI*fSSarR* to produce *farR*^G166D^ and *farR*^A14P^ variants; Cm^r^	This study
USA300 pGY*farE::lux*	USA300 with pGY*farE::lux* luciferase reporter vector; Cm^r^	(31)
USA300Δ*farR* pGY*farE::lux*	USA300Δ*farR* with pGY*farE::lux* luciferase reporter vector; Cm^r^	This study
USA300Δ*farR* pGY*farRE*::lux	USA300Δ*farR* with pGY*lux* vector containing *farR^xy^* gene and P_*farE*_ promoter segment driving luciferase reporter expression; *farR^xy^E* segment derived from USA300 (*farRE::lux*), FAR7 (*farR^H121Y^*), ST5 MRSA with *farR*^A14P^ or SDM of *pLIfarR* for *farR*^G166D^; Cm^r^	This study
H10388	FarR^A14P^ ST228 HA-MRSA Switzerland	(30)
H10497	FarR^A14P^ ST228 HA-MRSA Switzerland	(30)
H15532	FarR^A14P^ ST228 HA-MRSA Switzerland	(30)
H16035	FarR^A14P^ ST228 HA-MRSA Switzerland	(30)
H16125	FarR^A14P^ ST228 HA-MRSA Switzerland	(30)
H18341	FarR^A14P^ ST228 HA-MRSA Switzerland	(30)
H18412	FarR^A14P^ ST228 HA-MRSA Switzerland	(30)
H18583	FarR^A14P^ ST228 HA-MRSA Switzerland	(30)
H27777	FarR^G166D^ ST5 HA-MRSA United States	SAMN02383731
T89906	FarR^G166D^ ST5 HA-MRSA United States	SAMN02398797
W76127	FarR^G166D^ ST5 HA-MRSA United States	SAMN02383795
F29982	FarR^G166D^ ST5 HA-MRSA United States	SAMN02385374
F41882	FarR^G166D^ ST5 HA-MRSA United States	SAMN02383613
M018715	FarR^G166D^ ST5 HA-MRSA Netherlands	SAMN30843799
M019250	FarR^G166D^ ST5 HA-MRSA Netherlands	SAMN30843818
MPROS 0440	FarR^H121Y^ ST8 CA-MRSA United Kingdom	SAMEA1572448
MPROS 0385	FarR^H121Y^ ST8 CA-MRSA United Kingdom	(39)
*E. coli*		
DH5α	Transformation competent strain. λ^−^ϕ80d*lacZ*ΔM15 Δ(*lacZYA*-*argF*)*U169 recA1 endA1 hsdR17*(*r*_*K*_− *m*_*K*_−) *supE44 thi*-*1 gyrA relA1*	Invitrogen
Plasmid		
pKOR-1	*E. coli*-*S. aureus* shuttle vector; contains *Pxyl*/*tetO*; antisense *secY* RNA expression; Amp^r^, Cm^r^	(62)
pKOR*farR*	pKOR-1 containing ligated PCR products generated with primer pairs *farR*-UP-*att*B1/*farR*UP-*Sac*II, and *farR*-DW-*Sac*II/*farR*-DW-*att*B2	This study
pLI50	*E. coli-S. aureus* shuttle vector; Amp^r^, Cm^r^	(63)
pLI*farR*	pLI50 with native *farR* gene; Amp^r^, Cm^r^	(13)
pLI*farR*^H121Y^	pLI50 with *farR*^*H121Y*^ from *S. aureus* FAR7; Amp^r^, Cm^r^	(13)
pLI*farR*^G166D^	pLI50 with *farR*^G166D^ from SDM of pLI*farR* from *S. aureus* USA300; Amp^r^, Cm^r^	This study
pLI*farR*^A14P^	pLI50 with *farR*^A14P^ from SDM of pLI*farR* from *S. aureus* USA300; Amp^r^, Cm^r^	This study
pGY*lux*	*E. coli*-S. aureus shuttle vector harboring promoterless luxABCDE operon; Amp^r^, Cm^r^	(64)
pGY*farE*::*lux*	*farE* promoter segment cloned in pGY*lux*; *Amp*^*r*^*, Cm*^*r*^	(31)
pGY*farRE*::*lux*	pGYlux with native farR and PfarE promoter segment from USA300; Amp^r^, Cm^r^	(24)
pGY*farRE*::*lux* (FarR^H121Y^)	pGY*lux* with *farR* and P_*farE*_ promoter segment from *S. aureus* FAR7 (*farR*^H121Y^); Amp^r^, Cm^r^	(24)
pGY*farRE*::*lux* (FarR^G166D^)	pGY*lux* with *farR* and P_*farE*_ promoter segment from SDM of pLI*farR* from *S. aureus* USA300 (*farR*^G166D^); Amp^r^, Cm^r^	This study
pGY*farRE*::*lux* (FarR^A14P^)	pGY*lux* with *farR* and P_*farE*_ promoter segment from SDM of pLI*farR* from *S. aureus* USA300 (*farR*^A14P^); Amp^r^, Cm^r^	This study
pET*farR*	pET28 with *farR* from USA300; contains T7-IPTG-inducible promoter; Km^R^	This study
pET*farE*^sECD^	pET28 with two soluble extracytoplasmic domains from *farE* from USA300; contains T7-IPTG-inducible promoter; Km^R^	This study

^*a*^ SDM, site-directed mutagenesis.

### Strain and plasmid construction

Genetic manipulation of *S. aureus* was conducted following established guidelines (59) and as described in our previous work (13, 24, 31, 65). Restriction enzymes and T4 DNA ligase were purchased from New England BioLabs, Taq polymerase from GenScript, kits for PCR cleanup and plasmid preparation from Geneaid, and oligonucleotide primers (Table 3) from Integrated DNA Technologies. All plasmids for genetic manipulation of *S. aureus* were constructed as shuttle vectors in *E. coli* DH5α, and their integrity was confirmed by nucleotide sequencing of the cloned DNA fragments using the primers pLI50_F and pLI50_R for genes cloned in pLI50, pGYlux_F, and pGYlux_R for products cloned in pGY*lux,* and pKOR_F and pKOR_R for products cloned in pKOR-1. All shuttle vectors were then transformed by electroporation into *S. aureus* USA300 or isogenic derivatives using *S. aureus* RN4220 as an intermediate host.

**TABLE 3:** PCR primer sequences

Primer	Sequence^*a*^
pLI50_F	5′-ATTTCCCCGAAAAGTGCC-3′
pLI50_R	5′-TTTCTCGGCATAAATGCG-3′
pGY*lux*_F	5′-CTGTTGTTTGTCGGTGAACGCT-3′
pGY*lux*_R	5′-ATTGGGGAGGTTGGTATGTAAGC-3′
pKOR_F	5′-CAGATCCATATCCTTCTTTTTCTGA-3′
pKOR_R	5′-TGTGGAATTCTGAGCGGATA-3′
SDM-G166D_F	5′-GATTGGCCTGaCGAAGATATTGATAACATTTTCCATAG-3′
SDM-G166D_R	5′-CAATATCTTCGtCAGGCCAATCAATTTTTTCATC-3′
SDM-A14P_F	5-GTTATAAAGACAAAAAAAcCATTGTCGAGTAGCTTGCTACAATTG-3′
SDM-A14P_R	5′-CTGACTAAATGCTCAATATTTCTGTTTTTTTgGTAACAGCTCATCG-3′
*farRE*::*lux*_F	5′-CGATAGTAGTACACGgATcCATTAACGTGTACACTATCG-3′
*farRE*::*lux*_R	5′-CATTGTCAAATgTCGacGCATTTGTAGCAAGTGG-3′
farE-SD1_F	5′-GATACCACTTGCTAgcAATGCACCGAAATTTGAC-3′
farE-SD1_R	5′-CTGGAATACCAAGTCGCATATGACCCTACTTCTGTAGATGTC-3′
farE-SD2_F	5′-GACATCTACAGAAGTAGGCGGTCATATGCGACTTGGTATTCCAG-3′
farE-SD2_R	5′-GACTGAGGCAAATAAAGGgAGCTCCTaATTTAACTTTTTAGACATATC-3′
*farR*-UP-*attB1*^*b*^	5′-CTACCTGCTGTTCCTATTGC-3′
*farR*-UP-*Sac*I	5′-ttttgagctcAGTCTCTTTCATCTACATTTCTCCTTTGTGTG-3′
*farR*-DW-*Sac*I	5′-ttttgagctcTAGTAGATGAGAAACTCATGAGCGTTACC-3′
*farR*-DW-*attB2*^*c*^	5′-CGTCAGCATTTGGTTTTTCTGG-3′

^*a*^ Underlined lowercase nucleotides represent primer-directed changes for incorporation of nucleotide substitutions in site-directed mutagenesis (SDM-G166D, SDM-A14P) or 5′ additions for incorporation of *Sac*I sites (*farR*-UP-*Sac*I and *farR*-DW-*Sac*I, respectively).

^*b*^ *attB1 *GGGGACAAGTTTGTACAAAAAAGCAGGCT.

^*c*^ *attB2* GGGGACCACTTTGTACAAGAAAGCTGGGT.

Site-directed mutagenesis was used to PCR-amplify the pLI*farR* plasmid and mutate the nucleotide sequence of variants for which there was no clinical isolate currently available. The PCR primers SDM-G166D_F and SDM-G166D_R or SDM-A14P_F and SDM-A14P_R were used to generate *farR*^G166D^ and *farR*^A14P^, respectively. Plasmids were then digested with *Dpn*I prior to transformation of *E. coli* DH5α to create complementation plasmids in which *farR* is expressed from its native promoter, as previously described for wild-type *farR* and *farR*^H121Y^ (13). For luciferase reporter constructs, primers *farRE::lux*_F and *farRE::lux*_R were used to amplify a 905 bp product comprising *farR* and the adjacent P*_farE_* promoter segment, which was then digested with *Bam*HI and *Sal*I for ligation into the pGYlux reporter vector, such that luciferase expression is driven from the *farE* promoter under control of wild-type or variant *farR* (64).

USA300Δ*farR,* containing an in-frame, markerless deletion of *farR* (SAUSA300_2490), was generated using pKOR-1 (62). Briefly, ~1 kbp segments upstream and downstream of *farR* were amplified using the PCR primers *farR*-UP-*attB1* and *farR*-UP-*SacI* or *farR*-DW-*SacI* and *farR*-DW-*attB2* for the upstream and downstream segments, respectively. Segments were digested with *Sac*I, then ligated together for incorporation into pKOR-1 to generate pKOR*farR* using BP Clonase II (Invitrogen). To generate the in-frame, markerless deletion, USA300 pKOR*farR* underwent a two-step temperature shift and antisense counterselection, as previously described by Bae and Schneewind (62).

### Expression and purification of recombinant protein

Recombinant 6×His-FarR was expressed and purified from *E. coli* BL21. Bacterial cultures were grown at 37°C in LB (Sigma-Aldrich) and supplemented with 50 µg/mL kanamycin, to an optical density at 600 nm (OD_600_) of 0.3, before the addition of 0.2 mM IPTG and incubation at room temperature with shaking for an additional 18 h. Cells were prepared by harvesting by centrifugation at 4,200 × *g* and 4°C for 15 min and washed twice in 1× phosphate-buffered saline (PBS). Cells were then incubated with lysis buffer (150 mM NaCl, 50 mM Tris-HCl, pH 8.0, 1% [vol/vol] Triton X-100, 0.5% [vol/vol] SDS, 0.5% sodium deoxycholate) and supplemented with Pierce EDTA-free protease inhibitor cocktail, 10 µg/mL lysostaphin, and 1 U DNase I. Lysates were incubated for 1 h at room temperature with agitation, followed by centrifugation at 4,200 × *g* and 4°C for 15 min, after which the soluble fraction was filtered through a 0.45 µm Acrodisc syringe filter (Pall Laboratory). The lysate was applied onto a 1 mL HisTrap Cobalt affinity column equilibrated with binding buffer containing 50 mM sodium phosphate (pH 8). After washing extensively with binding buffer and wash buffer, bound His-tagged protein was eluted with up to 0.25 M imidazole in 50 mM sodium phosphate buffer (pH 8). Column fractions were assessed by SDS-polyacrylamide gel electrophoresis to check for purity, and fractions containing FarR protein were pooled and dialyzed in 20 mM sodium phosphate and 0.5 M NaCl (pH 7.4) at 4°C. Protein concentration was determined by Bradford assay using Bio-Rad protein assay reagent.

### Electrophoretic mobility shift assays

EMSAs were performed using recombinant 6×His-tagged FarR or variant derivatives, where the *farER* intergenic segment served as the DNA probe. Each EMSA reaction contained 50 ng oligonucleotide probe, up to 0.6 µM purified FarR, 240 µg/mL bovine serum albumin, and 15.2 µg/mL DH5α gDNA in EMSA buffer (20% glycerol, 30 mM Tris-HCl, pH 8.0, 1 mM MnCl_2_, 1 mM MgCl_2_, 120 mM KCl, and 16 mM dithiothreitol). Reaction mixtures were incubated at room temperature for 1 h, after which individual samples were applied to a 6% TBE-acrylamide gel and electrophoresed in 1× TBE at 120 V. Gels were stained in 1× TBE supplemented with 600 ng/mL ethidium bromide for 20 min and imaged using ChemiDoc Go (Bio-Rad).

### Growth and MIC assays

To prepare the inoculum for growth assays, single colonies of *S. aureus* clinical isolates or USA300 and USA300Δ*farR* harboring pLI50 or pLI*farR* variant derivatives were inoculated into 3 mL TSB in 12 × 75 mm polypropylene snap-cap tubes and grown overnight at 37°C on a rotary shaker. The OD_600_ of these overnight cultures was determined on a Beckman Coulter DU 530 spectrophotometer, and cultures were then seeded into fresh TSB to achieve an initial OD_600_ of 0.01. Growth assays were conducted in either flask cultures or microtiter plates, while testing for altered MIC was conducted in glass tubes. Variable concentrations of linoleic acid were employed as dictated by the strains that were being assessed and the mode of growth. For the assay of reporter gene expression in flask cultures, 20 µM linoleic acid was employed, since this is subinhibitory and does not significantly impair growth of USA300Δ*farR. S. aureus* is more tolerant of linoleic acid during growth in microtiter plates, under which condition we employed 50 µM linoleic acid to differentiate between wild-type and *farR*-deficient USA300, while 100 µM was employed in assays for enhanced resistance phenotypes. For growth assays, cultures were inoculated into 96-well microtiter plates containing 200 µL TSB + 0.1% dimethyl sulfoxide (DMSO) and the indicated concentrations of LA as detailed above. Growth of each culture was assessed in quadruplicate wells. Plates were incubated at 37°C in the Synergy H4 Reader with automated shaking, and optical density measurements were taken every 20 min for 24 h. As required, cultures were also inoculated in triplicate into 125 mL Erlenmeyer flasks containing 25 mL of TSB + 0.1% DMSO and indicated concentrations of linoleic acid, and samples were withdrawn at hourly intervals for OD_600_ determinations. For demonstration of enhanced resistance to linoleic acid through MIC assay, cells were subcultured to OD_600_ = 0.01 in triplicate 20 × 150 mm glass tubes with plastic caps containing 3 mL TSB + 0.1% DMSO and 800 µM linoleic acid, which is 2× MIC reported for wild-type USA300 (31). Tubes were placed on a rack at a 30° angle in a 37°C incubator with shaking at 220 rpm, and OD_600_ was measured after 24 h.

### Generation of FarE antibodies

FarE is a 90 kDa protein with 12 transmembrane-spanning helices, and two soluble extracytoplasmic domains bounded by transmembrane helices 1 and 2 and 7 and 8. DNA segments encoding the two soluble extracytoplasmic domains were amplified by PCR with primers *farE*-SD1_F and *farE*-SD1_R, and *farE*-SD2_F and *farE*-SD2_R, respectively (Table 3), and the separate SD1 and SD2 products were mixed in a 1:1 molar ratio in splicing overlap extension PCR using regions of overlap incorporated into the *farE*-SD1_R and *farE*-SD2_F primers, with Phusion polymerase and 200 µM dNTPs. After 15 cycles of denaturation at 95°C for 1 min, annealing at 50°C, and extension at 72°C for 2 min, outer primers *farE*-SD1_F and *farE*-SD2_R were added to the PCR mixture, followed by another 20 cycles (denaturation at 95°C for 1 min, annealing at 50°C, extension at 72°C for 2 min). After agarose gel electrophoresis, the fusion product was excised and cleaned via gel purification, digested with *NheI* and *SacI*, and ligated into pET28, creating pET*farE*, which was transformed into *E. coli* BL21 for protein expression and purification by metal affinity chromatography. Production of FarE-specific polyclonal antibodies in New Zealand White rabbits was contracted to ProSci Incorporated (Poway, CA). Each of two animals was initially immunized with 200 µg of 6×His-FarE emulsified in Freund’s adjuvant, followed by 100 µg of protein in incomplete Freund’s adjuvant, at 2 week intervals for 6 weeks. The animals were then bled for collection of antiserum 2 weeks after the final immunization.

### Western blotting

For preparation of cell lysates, *S. aureus* clinical strains harboring FarR variants, USA300, N315, USA300Δ*farR,* or USA300Δ*farE*, as well as USA300, USA300Δ*farE,* and USA300Δ*farR* harboring pLI50 and derivatives of FarR were grown to mid-exponential phase in TSB alone, or TSB + 20 µM LA. Cells were prepared by centrifugation at 4,200 × *g* and 4°C for 15 min and washed twice in 1× PBS. Cells were then incubated with lysis buffer (150 mM NaCl, 50 mM Tris-HCl, pH 8.0, 1% [vol/vol] Triton X-100, 0.5% [vol/vol] SDS, 0.5% sodium deoxycholate) and supplemented with Pierce EDTA-free protease inhibitor cocktail, 10 µg/mL lysostaphin, and 1 U DNase I. Lysates were incubated for 1 h at room temperature with agitation, followed by centrifugation at 4,200 × *g* and 4°C for 15 min. The clarified cell lysate was then assayed for determination of total protein by Bradford assay using Bio-Rad protein assay reagent to ensure normalization of protein loading. Samples containing 3–5 μg of total cell lysate protein were subjected to SDS-PAGE using a 12% polyacrylamide resolving gel. After transfer of proteins to the Amersham Hybond-P FluoroTrans polyvinylidene difluoride membrane (GE Healthcare) at 100 V for 1 h, the membranes were blocked by incubation in PBS supplemented with 5% non-fat skim milk powder. Wash buffer and antibody dilution buffer consisted of PBS plus 0.05% Tween 20, and PBS-Tween supplemented with 2% skim milk powder. Primary anti-FarE antiserum application overnight was followed by secondary IRDye 800-conjugated goat anti-rabbit IgG (Jackson ImmunoResearch Laboratories, Inc.). Membranes were imaged using an Odyssey CLx imager 32 (Li-Cor Biosciences). Densitometry analysis was performed using Fiji (ImageJ) (66, 67).

### Assays of reporter gene expression

For inducible expression, overnight inoculum cultures containing the appropriate reporter constructs were subcultured to OD_600_ = 0.01 into triplicate 125 mL flasks containing 25 mL of TSB + 0.1% DMSO and 20 µM linoleic acid, and then incubated at 37°C with orbital shaking (220 rpm) for 6 h. Samples (4 × 200 µL aliquots) were withdrawn at 1 h intervals or at the stationary endpoint (8 h), respectively, into 96-well white opaque flat-bottomed plates (Greiner Bio-One). Wells were supplemented with 20 µL of 0.1% (vol/vol) decanal in 40% ethanol, and luminescence measurements were immediately taken on a Synergy H4 hybrid reader (BioTek), with 1 s of integration and a gain of 200. Synchronously, OD_600_ was measured in triplicate with a spectrophotometer. Data values were recorded as relative light units (RLU), corrected for background by subtracting values recorded from cultures harboring empty pGYlux. Data points were standardized for differences in growth by dividing RLU values by the recorded OD_600_ values of the cultures when samples were withdrawn. For basal expression, overnight inoculum cultures containing the appropriate reporter constructs were subcultured to OD_600_ = 0.01 into triplicate 12 × 75 mm polypropylene snap-cap tubes containing 3 mL of TSB, then incubated at 37°C with orbital shaking (220 rpm) and grown to stationary phase, after which samples were removed for luminescence determination in microtiter plates as detailed above.

### Identification of FarR variant proteins and genotyping

As noted previously (24), the diversity of the FarR protein sequence across the phylogenetic spectrum of *S. aureus* comprised six primary clusters, of which cluster 2 is currently represented by 53,201 protein accessions across several common *S. aureus* clonal complexes, including ST5, ST8, ST15, and ST97. Current data for distribution of the C116Y, H121Y, G166D, and A14P variants in this cluster were obtained through BLAST homology searches for each single amino acid substitution variant, combined with use of the Identical Proteins link at NCBI. Multilocus sequence typing was done by scanning genome assemblies against the MLST database (https://pubmlst.org/) using the MLST 2.0 program, selecting for *S. aureus* (68). Staphylococcal protein A gene (spa) typing was conducted using spaTyper 1.0 (69). Typing of SCC*mec* was conducted using SCCmecFinder 1.2 from a curated database (https://cge.food.dtu.dk/services/SCCmecFinder/). Determination of resistance genes and antimicrobial resistance was conducted using ResFinder 4.6.0 with default settings and selection of *S. aureus* (70, 71) or from analyzing genome assemblies from NCBI (https://www.ncbi.nlm.nih.gov/datasets/genome/).

### Emergence of FarR variants in the phylogenetic spectrum of ST97 *S. aureus*

For this purpose, completed genomes of ST97 *S. aureus* from both human and bovine hosts were selected from the Pathosystems Resource Integration Center database (32). *Staphylococcus aureus* genome assemblies from 75 isolates and the *S. aureus* USA300_FPR3757 reference genome were analyzed using the Bactopia pipeline (72), where assemblies were converted into 250 bp Illumina reads with a minimum PHRED score of Q33 and subsequently mapped to the *S. aureus* USA300_FPR3757 reference genome (NC_007793.1) through Snippy-core (v.4.6.0) using default parameters (33). A core SNP alignment was generated using Snippy-core and used to construct a maximum-likelihood phylogeny with IQ-TREE (v.2.2.2.7) using an ultrafast bootstrap replicate of 1,000 and the ModelFinder parameter to ensure the automatic selection of the best-suited model (34). SNP distances were computed from the pairwise differences between the core genome alignment using snp-dists (73), and the tree was rooted using the USA300_FPR3757 genome as an outgroup. Data were visualized and presented with RStudio (74).

### Statistical analyses

Data points for growth, MIC, and reporter gene assays were plotted and analyzed using GraphPad Prism version 10. Significant differences at specific time points were determined using GraphPad statistics functions, including one-way ANOVA, two-way ANOVA, and Tukey’s test for multiple comparisons.

## SUPPLEMENTAL MATERIAL

Supplemental figures spectrum.02278-25-s0001.pdf Fig. S1 and S2.

Supplemental tables spectrum.02278-25-s0002.xlsx Tables S1 to S8.

ASM does not own the copyrights to Supplemental Material that may be linked to, or accessed through, an article. The authors have granted ASM a non-exclusive, world-wide license to publish the Supplemental Material files. Please contact the corresponding author directly for reuse.
