# Supplementary material for: Widespread emergence of Staphylococcus aureus with variant FarR regulators and enhanced resistance to antimicrobial fatty acids within clonal complex CC5, CC8, and CC97 strains from human and bovine hosts
Source: Microbiol Spectr. 2025 Oct 31;13(12):e02278-25. doi: 10.1128/spectrum.02278-25 (PMC12671165; doi:10.1128/spectrum.02278-25)
Supplement: Supplemental figures — Fig. S1 and S2. [file spectrum.02278-25-s0001.pdf]

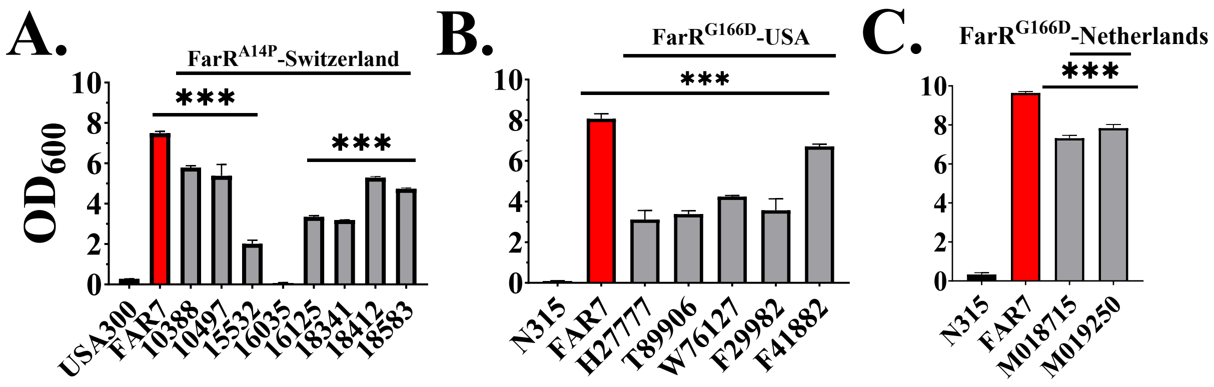

FIG S1. Twenty-four hour endpoint growth of FarR variant strains demonstrating enhanced linoleic acid resistance. Cultures of USA300, N315, FAR7 or MRSA harboring $FarR^{A14P}$ from Switzerland (A) or $FarR^{G166D}$ from either the United States (B) or the Netherlands (C) were inoculated to $OD_{600} = 0.01$ into triplicate tubes containing 3 mL of TSB supplemented with 800 μM LA + 0.1% DMSO. Tubes were incubated at 37 °C with orbital shaking, and growth (OD_{600}) was determined after 24 hours. Each data point represents the mean ± standard error of the mean (SEM) from triplicate cultures. Statistically significant differences (***, $P < 0.001$; *, $P < 0.05$) compared to USA300 or N315 were determined by Tukey's multiple-comparison test.

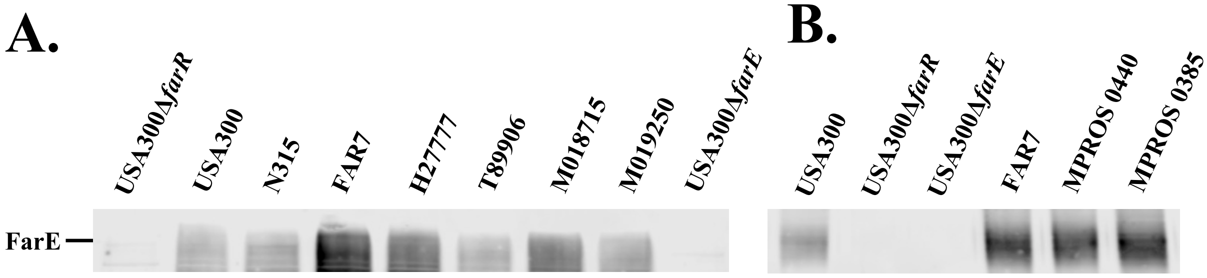

FIG S2. Clinical isolates harbouring FarR variants promote enhanced production of FarE. Western blot gels loaded with 5 μg of cell lysate protein from (A) $FarR^{G166D}$ or 3 μg of cell lysate protein from (B) $FarR^{H121Y}$. Blots were probed with FarE antisera. Protein concentration normalized using Bradford Assay.
